# The effect of the COVID-19 pandemic on epistaxis and anaemia in patients with hereditary haemorrhagic telangiectasia (HHT) in central South Africa

**DOI:** 10.1186/s13023-025-03777-2

**Published:** 2025-07-16

**Authors:** Stephanie Juané Kennedy, Leriska Haupt, Riaz Yakoob Seedat

**Affiliations:** 1 Department of Haematology and Cell Biology, Faculty of Health Sciences, University of the Free State, Bloemfontein, South Africa; 2 National Health Laboratory Service, Universitas Academic Laboratory, Bloemfontein, South Africa; 3 Department of Otorhinolaryngology, Faculty of Health Sciences, University of the Free State, Bloemfontein, South Africa; 4 Department of Otorhinolaryngology, Universitas Academic Hospital, Bloemfontein, South Africa

**Keywords:** COVID-19, Epistaxis, Hereditary haemorrhagic telangiectasia, Masks, South Africa

## Abstract

**Background:**

Recurrent epistaxis, which frequently results in iron deficiency anaemia and impaired quality of life, is the most frequent complication of hereditary haemorrhagic telangiectasia (HHT). Specific data to guide rare disease management during a pandemic such as COVID-19 are lacking.

**Methods:**

To better define the impact of the COVID-19 pandemic on HHT, we conducted a retrospective and prospective observational descriptive review of HHT patients in central South Africa. Epistaxis severity scores (ESSs) and haemoglobin (Hb) levels before and after the start of the pandemic were compared. Variables that may have influenced epistaxis severity and anaemia were investigated, viz. (i) mask use, (ii) nasal versus oral swabs to test for SARS-CoV-2, (iii) COVID-19 disease and (iv) its management, (v) COVID-19 vaccines, and (vi) the social impact of the pandemic.

**Results:**

Twenty-four patients with confirmed HHT were included in the study. Subset analyses revealed a clinically significant change in ESSs (≥ 0.71 minimal important difference) and Hb levels (> 2.7% biologic variation) in 6/11 (54.6%) and 12/15 (80%) patients. While the median ESS improved in 2/11 (18.2%) patients, it worsened in 4/11 (36.4%) patients during the pandemic. However, the changes in the median ESS (2.25 pre-pandemic versus 2.5 during the pandemic; p = 0.38) and Hb level (9.5 g/dL pre-pandemic versus 10 g/dL during the pandemic; p = 0.38) for the study population were not statistically significant. Clinical and social variables that may influence epistaxis severity and anaemia were identified. Nasal swab testing for SARS-CoV-2 induced epistaxis in 9/12 (75%) cases and was noted as an important factor.

**Conclusion:**

The COVID-19 pandemic has had a clinically significant impact on epistaxis severity and anaemia in some individuals with HHT in central South Africa. Specific strategies are needed to optimise the management of HHT during the COVID-19 and future respiratory pandemics.

## Introduction

Hereditary haemorrhagic telangiectasia (HHT) is a heterogeneous autosomal dominant disorder of angiogenesis with an estimated prevalence of 1:5000 to 1:10,000 [1–3]. HHT results from pathogenic variants in one of four genes (*ENG, ACVRL1, SMAD4, GDF2*) that encode proteins in the bone morphogenetic protein (BMP)/transforming growth factor-beta (TGF-beta) signalling pathway [3, 4]. Clinically, this results in mucocutaneous telangiectasias and visceral arteriovenous malformations (AVMs) [5]. Recurrent epistaxis, which frequently results in iron deficiency anaemia and impaired quality of life, is the most frequent complication of HHT [1].

Although HHT is a rare disease, it deserves special attention during a pandemic like COVID-19 [6]. The COVID-19 pandemic posed unique clinical and social challenges in patients with HHT. A few studies, mainly in Italy and Spain, investigated these challenges by focusing on prevalence, management, testing methods, mental health and quality of life, and virtual consultations [6–11]. There is a lack of research on the impact of COVID-19 on HHT patients in low and middle-income countries.

This study aimed to evaluate the impact of the COVID-19 pandemic on patients with HHT in the Free State and Northern Cape provinces of South Africa. The objectives of the study were: (1) to compare epistaxis severity scores (ESSs) and haemoglobin (Hb) levels in patients with HHT before and during the pandemic, (2) to investigate factors that may have influenced epistaxis severity and anaemia during the COVID-19 pandemic, including facemask use, nasal versus oral swabs to test for SARS-CoV-2, COVID-19 disease and its management, COVID-19 vaccines, and the social impact of the pandemic.

## Methods

### Study design

This study used an explanatory mixed-method study design, with collection of quantitative and qualitative data of patients with HHT attending the Universitas Academic Hospital Bleeding Disorders Clinic (UHBDC), Bloemfontein, Free State, South Africa, during the COVID-19 pandemic.

Patients with HHT in central South Africa (mainly the Free State and Northern Cape provinces) are managed at the UHBDC.

### Participants

All patients with confirmed HHT (meeting molecular or Curaçao criteria) on the UHBDC records were included. To compare Hb levels and ESSs before and during the pandemic, inclusion and exclusion criteria were specified for these subset analyses.

The date on which the World Health Organization (WHO) declared COVID-19 a pandemic, 11 March 2020 [12], was taken as the start of the pandemic. The study period started six months before the pandemic and ended on 26 January 2023. Only patients who attended the UHBDC at least once between 11 September 2019 and 10 March 2020 and at least once after the start of the pandemic, were included in the subset analyses.

### Measurement

Data were collected from patient files and the hospital information system. Where information from the clinical records was unclear, patients were asked for clarification during their routine clinic visits or by telephone. The National Health Laboratory Service laboratory information system (NHLS LIS) was used to verify laboratory results, viz., Hb levels and reverse transcriptase polymerase chain reaction (RT-PCR) tests for SARS-CoV-2.

The ESS is a semi-objective, validated scoring system for the severity of epistaxis in HHT [13]. ESSs that were documented in patient notes were confirmed using an online ESS calculator [14]. Where the ESS was not documented, it was calculated using data involving epistaxis frequency, duration, intensity, need for medical attention, and transfusion, as recorded in the clinical notes. The ESS was omitted if it could not be calculated due to missing data sets. Hb levels were obtained from the NHLS LIS and used to confirm the presence or absence of anaemia for the ESS calculation. WHO guidelines were used to define anaemia [15].

Patients were asked if nasal or oral swabs were performed and whether nasal swabs induced epistaxis. SARS-CoV-2 PCR results for all patients were recorded, and disease severity and management data were collected for patients with positive results. Patients were asked if they noticed any effect on epistaxis severity with facemask use, following vaccination, or due to heightened emotional stress levels during the pandemic. Vaccine hesitancy and its underlying reasons were recorded. Where patients elected to defer clinic appointments due to fears of COVID-19 exposure at a healthcare facility, or missed clinic appointments due to transport restrictions during lockdown, this was recorded.

### Statistical analysis

Categorical variables were reported as numbers and percentages. Quantitative variables were described as medians. A two-sided Student t-test for paired data was used, and a p-value of less than 0.05 was considered statistically significant. A change of 0.71 in intra-individual ESSs (the minimal important difference in ESS as determined by Yin et al. [16]) was considered clinically significant. Intra-individual biologic variation of 2.7% was used to assess the clinical significance of a change in Hb [17].

## Results

**Fig. 1:**
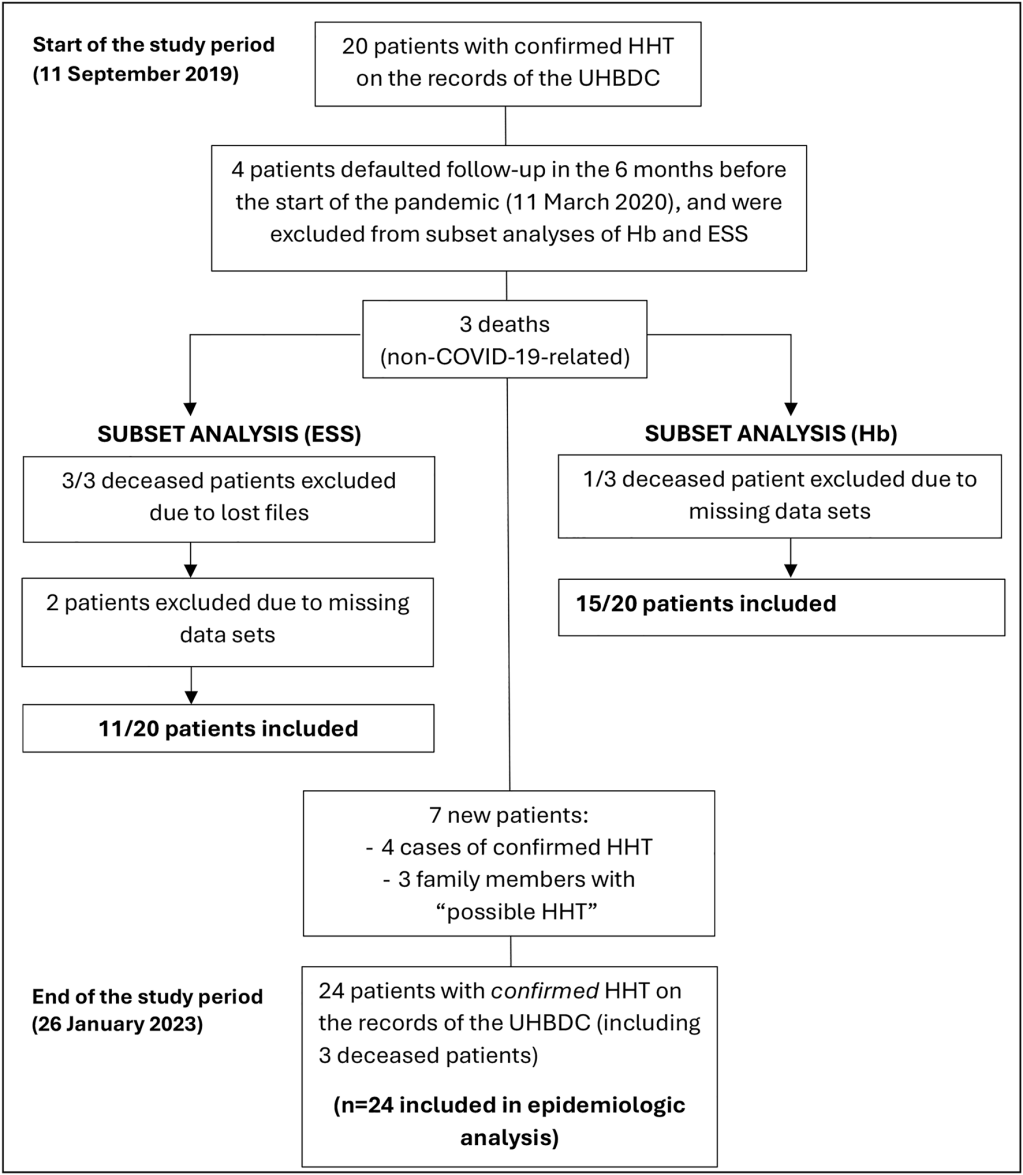
Patient selection flow diagram. *COVID-19* coronavirus disease 2019; *ESS* epistaxis severity score; *Hb* haemoglobin; *HHT* hereditary haemorrhagic telangiectasia; *UHBDC* Universitas Academic Hospital Bleeding Disorders Clinic

Twenty-four patients were included for epidemiologic data analysis (Fig. 1). Fifteen and 11 patients (patients nos. 1–15 and 1–11) were eligible for inclusion in the subset analyses of Hb levels and ESSs, respectively. Patients nos. 16–24 were excluded from the subset analyses due to missing data points. Patients who defaulted follow-up in the six months before the pandemic (n = 4) and patients who were newly diagnosed after the pandemic started (n = 4), were excluded. Two of the three patients who died during the study period were still attending the clinic during the first 12 months of the study. Their clinic files were lost, so their ESSs could not be included. However, their Hb levels were retrievable from the NHLS LIS and were included. Two further patients were excluded from ESS analysis due to missing data sets. However, their laboratory data were available for inclusion in Hb analysis.

### Baseline characteristics and SARS-CoV-2 PCR tests

The baseline characteristics of study participants (n = 24) and their SARS-CoV-2 PCR tests (type of swab, result, and nasal swab-induced epistaxis or not) are illustrated in Table 1.

**Table 1:** Baseline characteristics and SARS-CoV-2 PCR tests (type of swab, result, and nasal swab-induced epistaxis or not)

Patient no.	Age (years)	Sex	Comorbid conditions	Genetic variant	Recurrent epistaxis	Muco-cutaneous telangiectasias	TTCE: Grade of right to left shunt	AVM(s)	SARS-CoV-2 PCR type of swab/result	Nasal swab-induced epistaxis
1	69	M	Migraine, Allergic rhinitis, Atopic dermatitis, BPH, Aspirin and NSAID use*	*ENG*	Y	Y	1	Nil	Oral swab/Negative	NA
2	42	M	HIV*, Allergic rhinitis, Atopic dermatitis	*ENG*	Y	Y	1	GI telangiectasias*, PAVM	Not done	NA
3	65	F	HT, Asthma, Allergic rhinitis, Atopic dermatitis	*ENG*	Y	Y	2	Nil	Nasal swab/Negative	Y (required nasal packing)
4	37	F	HMB*, Portal HT with splenomegaly	*ENG*	Y	Y	2	PAVM, Pancreatic AVM	Not done	NA
5	41	M	HIV*	*ENG*	Y	Y	1	Nil	Nasal swab/Negative	N
6	48	F	Right hemiparesis (previous stroke due to CAVM), Migraine, Smoking	*ENG*	Y	Y	1	CAVM	Oral swab/Negative	NA
7	27	F	HT, Pregnancy*	*ENG*	Y	Y	1	Nil	Nasal swab/Negative	Y
8	41	F	HMB*	Unknown	Y	Y	1	Nil	Nasal swab/Negative	Y
9	15	F	Nil	Unknown	Y	Y	2	Nil	Not done	NA
10	17	M	Nil	Unknown	Y	Y	†	†	Not done	NA
11	38	F	Pregnancy* with IUD at 29 weeks following a major nosebleed	Unknown	Y	Y	†	HAVM	Oral swab/Negative	NA
12	67	M	HT, Asthma, Previous PE, Chronic epidural haemorrhage post head injury	*ENG*	Y	Y	0	Nil	Nasal swab/Negative	Y
13	67	F	HIV*, Ankle osteoarthritis	*ENG*	Y	Y	†	†	Not done	NA
14	46	M	HIV*, Post-tuberculosis empyema with restrictive lung disease and cor pulmonale*	*ENG*	Y	Y	0	Nil	Nasal swab/Positive	Y
15	68	F	Cervix carcinoma*	*ACVRL1*	Y	Y	0	CAVM, HAVM	Not done	NA
16	36	F	Pregnancy*	*ACVRL1*	Y	Y	0	Nil	Nasal swab/Negative	Y
17	57	M	Nil	Unknown	Y	Y	†	GI telangiectasias*	Nasal swab/Negative	N
18	37	F	Graves’ disease*, HT, Headaches, NSAID use*	Unknown	Y	Y	†	†	Nasal swab/Negative	Y
19	49	F	Gastritis	Unknown	Y	Y	†	†	Oral swab/Negative	NA
20	20	M	Nil	*ENG*	Y	Y	3	Nil	Not done	NA
21	47	F	HIV*	*ENG*	Y	Y	3	Nil	Not done	NA
22	43	F	Deceased	*ENG*	Y	Y	†	†	Nasal swab Negative	Y
23	32	M	Nil	*ENG*	Y	Y	†	†	Nasal swab/Positive	N
24	52	M	Nil	Unknown	Y	Y	†	†	Nasal swab/Negative	Y

*ACVRL1* activin A receptor like type 1, *AVM* arteriovenous malformation, *BPH* benign prostatic hyperplasia, *CAVM* cerebral arteriovenous malformation, *ENG* endoglin, *GI* gastrointestinal, *HAVM* hepatic arteriovenous malformation, *HIV* human immunodeficiency virus, *HMB* heavy menstrual bleeding, *HT* hypertension, *IUD* intrauterine death, *NSAID* non-steroidal anti-inflammatory drugs, *PAVM* pulmonary arteriovenous malformation, *PCR* polymerase chain reaction, *PE* pulmonary embolism, *TTCE* transthoracic contrast echocardiography, *M* male, *F* female, *Y* yes, *N *no, *NA* not applicable

*Indicates confounding variables that could affect anaemia.

^†^Indicates incomplete workup for AVMs.

### Epistaxis severity and anaemia

**Fig. 2:**
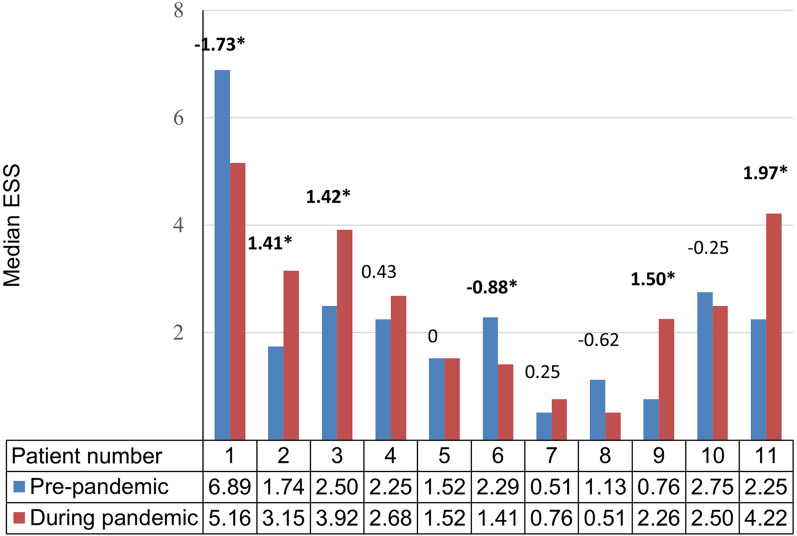
Individual median Epistaxis Severity Score (ESS) before and during the COVID-19 pandemic. Individual median ESSs before and during the COVID-19 pandemic for patients 1 to 11 are compared. A minimal important difference ≥ 0.71 [16] was considered a clinically significant change in ESS. An asterisk indicates those patients where the minimal important difference increased or decreased by ≥ 0.71.

The change in the median ESS was clinically significant (≥0.71) in 6/11 (54.6%) patients during the pandemic when compared with their pre-pandemic ESSs (Fig. 2). The median ESS decreased by ≥ 0.71 in 2/11 (18.2%) patients, while it increased by ≥ 0.71 in 4/11 (36.4%) patients during the pandemic. It remained unchanged in 5/11 (45.5%) patients.

Seven of the 11 patients included in the ESS subset analysis (nos. 1–7) had an *ENG* variant, while the variants in the other four were unknown. The sample size was too small to draw conclusions about genotype–phenotype correlations. However, the median ESS variably improved in 2/7 (28.6%), worsened in 2/7 (28.6%), and remained unchanged in 3/7 (42.8%) of the patients with *ENG* variants, which suggests that external or environmental factors influenced these changes during the pandemic.

**Fig. 3:**
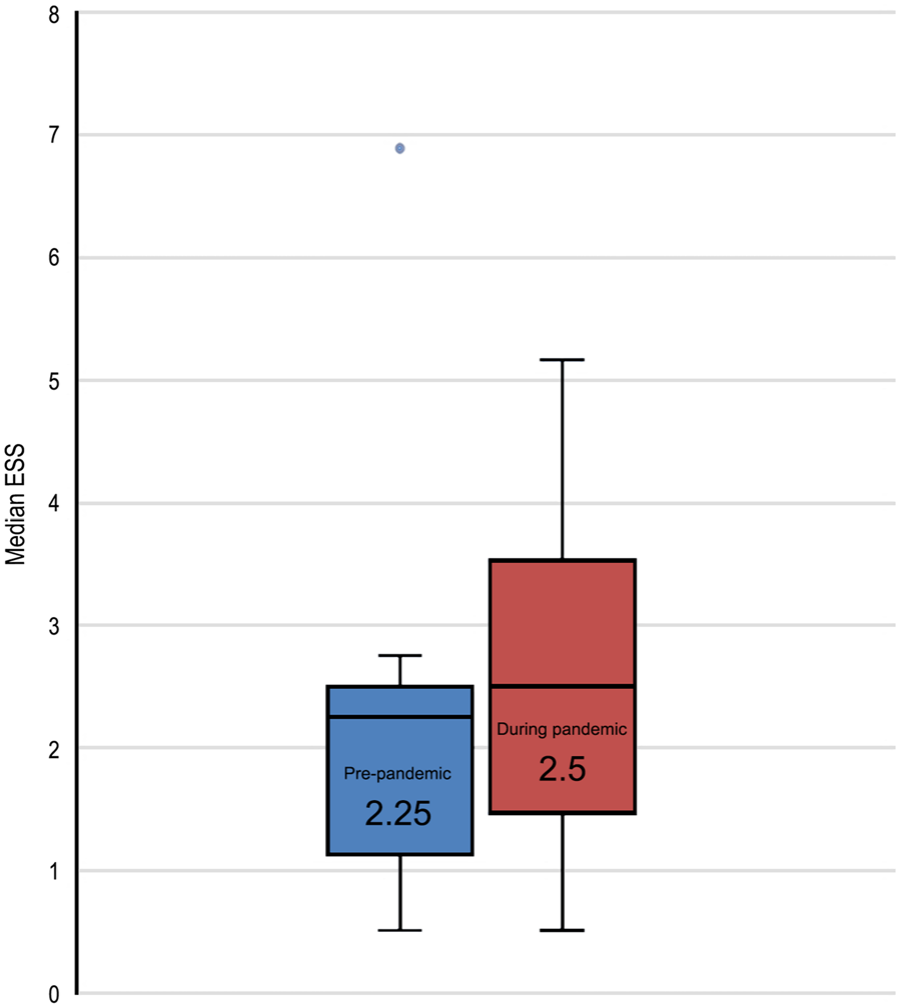
Median Epistaxis Severity Score (ESS) for the cohort (n = 11, patients 1–11) before and during the COVID-19 pandemic.

The change in the median ESS for the cohort during the pandemic was not statistically significant compared to the median ESS before the pandemic (2.25 versus 2.5; p = 0.38) (Fig. 3).

**Fig. 4:**
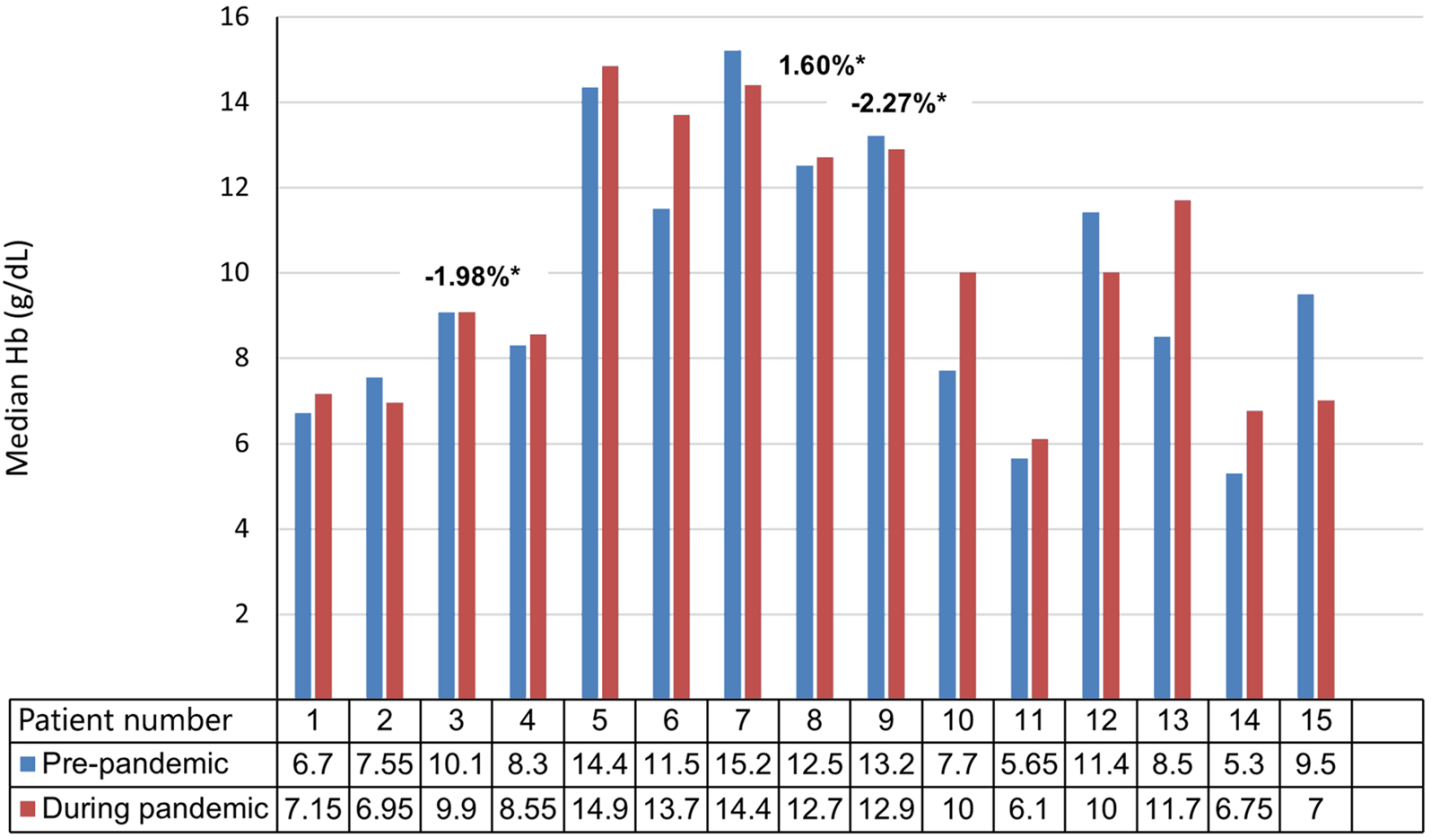
Individual median haemoglobin (Hb) level before and during the COVID-19 pandemic. Individual median Hb levels before and during the COVID-19 pandemic for patients 1-15 are compared. Normal biologic variation of < 2.7% [17] was considered a clinically *insignificant* change in Hb level. An asterisk indicates those patients where the change in median Hb was < 2.7%, indicating normal biologic variation. The change in median Hb exceeded 2.7% in the other patients and was considered clinically significant.

**Fig. 5:**
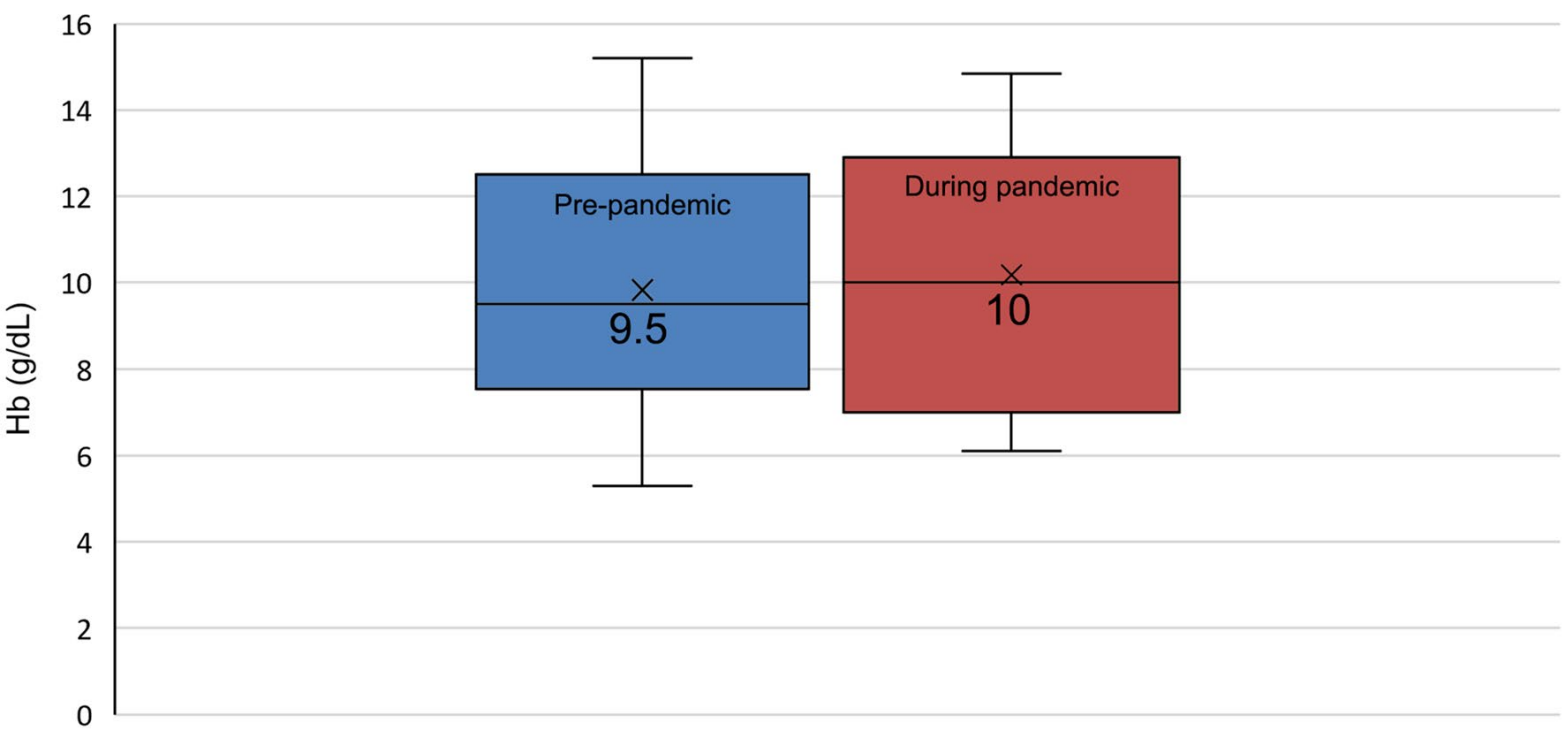
Median haemoglobin (Hb) level for the cohort (n = 15; patients 1–15) before and during the COVID-19 pandemic.

Of the patients included in the subset analysis of Hb levels, 11/15 (73.3%) and 10/15 (66.7%) patients were anaemic before and during the pandemic, respectively (Fig. 4). The median Hb level decreased in 6/15 (40%) and increased in 9/15 (60%) patients during the pandemic when compared with pre-pandemic values (Fig. 4). Intra-individual Hb changes exceeded biologic variation (2.7%) in 12/15 (80%) patients. The 0.5 g/dL increase in the median Hb level during the pandemic was not statistically significant compared to the median Hb level before the pandemic (9.5 g/dL versus 10 g/dL; p = 0.38) (Fig. 5).

Iron deficiency was confirmed as a cause of anaemia in all cases (defined as a ferritin level of <15 ng/mL documented at any point in the diagnostic workup), but comorbidities may have confounded Hb levels in some patients. These variables are indicated with an asterisk in Table 1. Other causes of anaemia included chronic disease (five patients had human immunodeficiency virus [HIV]), cancer (n = 2), pregnancy (n = 2), and other sources of blood loss such as gastrointestinal (GI) telangiectasias (n = 2) and heavy menstrual bleeding (HMB) (n = 2). Patient no. 1 also self-medicated with non-steroidal anti-inflammatory drugs (NSAIDs) and aspirin.

A subgroup analysis was conducted to determine the effect of these confounding variables on Hb levels. Two thirds (10/15) of the patients included in the Hb subset analysis (nos. 1, 2, 4, 5, 7, 8, 11, 13, 14, 15) had comorbidities that may have contributed to anaemia. A third (5/15) of the patients (nos. 3, 6, 9, 10, 12) did not have any additional variables that could influence their Hb levels. A clinically significant decrease in the median Hb level occurred in 4/15 (26.7%) patients (nos. 2, 7, 12, 15) during the pandemic. In this subgroup, 3/4 patients had other conditions that may have contributed to anaemia, including HIV and GI telangiectasias (no. 2), pregnancy (no. 7), and cervix carcinoma (no. 15). A clinically significant increase in the median Hb level occurred in 8/15 (53.3%) patients (nos. 1, 4, 5, 6, 10, 11, 13, 14) during the pandemic. This improvement occurred despite the presence of confounding conditions in seven of the eight patients in this subgroup. Packed red cell transfusions explained the increased Hb levels observed in patients 1 and 14 during the pandemic. However, despite the occurrence of HMB in patients 4 and 8, HIV in patients 5 and 13, and pregnancy in patient 11, the median Hb of these five patients improved during the pandemic. From this subgroup analysis we concluded that comorbidities may have contributed to anaemia in some patients (3/15; 20%), but that in the majority (12/15; 80%), other factors had a more significant influence on their Hb levels during the pandemic.

Further analysis revealed these factors to be epistaxis severity, transfusions, and improved pharmacologic management of bleeding and iron deficiency. In the 2/11 (18.2%) patients (nos. 2 and 7) whose median Hb level decreased by >2.7% during the pandemic, a parallel increase in median ESS (≥0.71) was observed. Likewise, in 3/11 (27.3%) patients (nos. 1, 6, 10) an increased median Hb level (>2.7%) was accompanied by a parallel decrease in median ESS (≥0.71) during the pandemic. However, in patients 4 and 11, an increase in median Hb was observed despite an increase in epistaxis severity, and in patient 5, the Hb increased while the ESS remained unchanged. These patients did not receive blood transfusions, and these contradictory observations are best explained by the fact that the updated 2020 international HHT guidelines [1] were implemented in the UHBDC during the pandemic, and the optimisation of IV iron infusions probably improved anaemia in these patients.

Five of the 24 (20.8%) patients required transfusion for symptomatic anaemia during the pandemic. Only two of these patients (nos. 1 and 14) were eligible for inclusion in the Hb subset analysis. Patient no. 1 required hospital admission, nasal packing, and blood transfusions following episodes of acute blood loss of up to 1.5 L with haemorrhagic shock.

Sixteen of the 24 patients (66.7%) were tested for SARS-CoV-2, and 2/16 (12.5%) cases had a positive PCR test, giving a prevalence of PCR-confirmed COVID-19 of 2/24 (8.3%) for the entire cohort. Patient no. 23 had mild COVID-19 disease with upper respiratory tract symptoms. He did not notice any change in his epistaxis. Patient no. 14 had severe COVID-19 pneumonia requiring high-flow oxygen. Despite uncontrolled HIV infection and a post-tuberculosis organised empyema complicated by cor pulmonale, he recovered and was discharged after ten days in hospital. The effect of COVID-19 on his ESS was not available since his file was lost. COVID-19 did not significantly impact his Hb level compared to his median Hb level during the pandemic (6.70 g/dL versus 6.75 g/dL; 0.75% variation). However, transfusion during the pandemic may have confounded his median Hb level. He passed away during the study period, but his death was not related to COVID-19.

Nasal swabs were performed in 12/16 (75%) patients, and oral swabs in 4/16 (25%). Nasal swabs induced epistaxis in 9/12 (75%) cases. One patient (no. 3) required nasal packing. This patient and several others reported that healthcare workers refused their requests for oral rather than nasal swabs.

Subjective reports on the effect of masks on epistaxis were available for 18 patients. Ten of the 18 patients (55.6%) noticed worsened epistaxis, while none reported improvement. ESS data was available for seven patients who felt that masks worsened their epistaxis. Only two of these patients had a significantly worse ESS during the pandemic than before the pandemic.

The majority (6/10; 60%) reported that they preferred medical masks to cloth masks because it was easier to breathe through medical masks, and cloth masks easily became blood-soaked. However, no patient noticed differences in the severity of epistaxis between cloth and medical masks. One of the ten patients (10%) preferred to wear a visor.

The vaccination status of 18 patients were known: 14/18 (77.8%) patients were vaccinated, and 1/14 (7.1%) received a booster dose. Eight of the 14 vaccinated patients (57.1%) received adenovector vaccines (Johnson & Johnson®), and 6/14 (42.9%) patients received mRNA vaccines (Pfizer®). Four of the 18 (22.2%) patients declined vaccination. Only one patient (1/18; 5.6%) expressed concerns about vaccination safety in a bleeding disorder. No patient reported any positive or negative effects on epistaxis following vaccination.

Data on the impact of the pandemic on clinic attendance was available for 18 patients. During the first months of the pandemic, 9/18 (50%) patients elected to defer their appointments due to fears of exposure to COVID-19 at healthcare facilities.

Restrictions on the number of passengers allowed in public transport vehicles during lockdown, resulted in 6/18 (33.3%) patients missing appointments. These patients all had refractory iron deficiency anaemia and required at least monthly iron infusions. The effect of missed appointments was reflected in their reduced Hb levels (>2.7% biologic variation) upon return to the clinic.

Data on the effect of emotional stress during the pandemic were available for 18 patients. Five of the 18 (27.8%) patients mentioned that they experienced heightened stress levels during the pandemic and that this exacerbated their epistaxis. A 12-year-old girl was sent home from school when her nose bled and soaked her mask. She was told that she might have COVID-19 and would infect her peers. This patient expressed feelings of embarrassment and marginalisation because of her rare disease.

## Discussion

This is the first study to report on the impact of the COVID-19 pandemic on patients with HHT in a low- or middle-income country and on the African continent. It provides valuable epidemiological data on SARS-CoV-2 infection in patients with a rare disease and highlights some of their unique clinical and social challenges.

Despite its small sample size, this study adds to data from other countries, which suggested that patients with HHT did not have an increased risk of COVID-19 infection or severe disease when compared with the general population [7–9].

The COVID-19 pandemic had a clinically significant impact on epistaxis severity and anaemia in several individuals with HHT in central South Africa. Based on our findings, we were able to suggest attention to aspects that may influence epistaxis severity in HHT patients during the COVID-19 and future respiratory pandemics (Table 2).

**Table 2:** Recommendations that may minimise epistaxis and anaemia in HHT patients during COVID-19 and future pandemics.

The risks and benefits of facemasks should be considered.
Frequent nasal moisturisation may reduce dry mucous membranes when masks are worn.
HHT patients should refrain from unnecessary nasal manipulation to adjust masks.
A visor may be better than no mask in HHT patients who cannot tolerate facemasks.
Oral rather than nasal swabs should be performed in HHT patients to test for SARS-CoV.
A card with written information about HHT may be helpful, as frontline healthcare workers may not be aware of the complications of this rare disease.
HHT patients should follow the same recommendations for COVID-19 vaccination as the general public.
We provide anecdotal evidence that stress may precipitate epistaxis in some patients. Continued emotional support is essential during the pandemic.

We were unable to demonstrate a statistically significant change in ESSs or Hb levels for the cohort during the pandemic. Several factors may have contributed to this lack of statistical significance, including the limited sample size, and the inconsistent improvement and worsening of these parameters observed at the individual level. These individual variations may be attributed to the variable expression of epistaxis severity inherent to the disease, as well as differences in the management of patients during the pandemic. The implementation of the updated 2020 international HHT guidelines [1] which coincided with the start of the pandemic, was an important factor that likely contributed to improvement in individual ESSs and Hb levels. However, where lockdown restrictions affected clinic attendance and adherence to treatment, the effect of interventions such as tranexamic acid prophylaxis and IV iron was not evident. In addition, transfusions led to increased Hb levels in two individuals during the pandemic, masking the severity of their anaemia, and affecting the statistical analysis of the cohort.

We identified several pandemic-related clinical and social factors that may affect epistaxis severity and anaemia in patients with HHT. Some patients felt that facemasks precipitated epistaxis. Several mechanisms may explain how masks could worsen epistaxis:

- Altered airflow in the nostrils could dry out mucous membranes. This may be particularly relevant in the dry central South African climate. Dry nasal mucosa is the most important precipitating factor for epistaxis in HHT, and nasal moisturisation is, therefore, the first line of treatment [1, 5].
- Adjusting the mask over the nose may cause mechanical trauma to the fragile telangiectasias.
- Masks may cause end-expiratory pressure that affects the pressure in the nasal vasculature.

The ideal mask in HHT would require minimal adjustment and absorb minimal moisture, while providing adequate protection against viral transmission.

Notably, the ESS was not significantly worse in most patients who felt masks aggravated their epistaxis. This suggests that these were probably subjective perceptions based on their masks being soaked with blood. Suppressa et al. [9] similarly concluded that mask use did not affect the bleeding profile in HHT patients.

Mask use may have additional benefits in patients with HHT. Their outdoor use could potentially delay the development of telangiectasias on the lips by reducing sun exposure. Geisthoff et al. [18] demonstrated that cutaneous telangiectasias in patients with HHT are more likely to occur in sun-exposed areas, including the face and lower lip. When considering the "second-hit" hypothesis in HHT, the inflammation associated with infections such as SARS-CoV-2 may also be a potential trigger for the development of vascular lesions [18]. However, these benefits need to be weighed against the potential of masks to trigger epistaxis in certain patients.

Nasal swabs should be avoided in HHT [19, 20]*.* Matti et al. [20] described an alternative procedure of nasopharyngeal swab collection through the transoral route. Repeat oral swabs to test for SARS-CoV-2 can be done to overcome their reduced sensitivity compared with nasal swabs [19]. Adherence to these recommendations and increased awareness of HHT may have prevented the nasal swab-induced epistaxis that patients experienced in this study. Unfortunately, some healthcare workers ignored patients’ warnings that nasal swabs could cause epistaxis. These practitioners were probably unaware of HHT, as it is underdiagnosed and underreported [1, 18].

Epistaxis following COVID-19 vaccination has been reported in the general population and is more frequently associated with adeno-vectored than mRNA vaccines [21]. The mechanisms that lead to bleeding are not well elucidated [21]. No patient in our study reported any effect on epistaxis following vaccination, but the small number of vaccinated patients limited these results. Of the four patients who refused vaccination, only one expressed concern about vaccination safety in a bleeding disorder. Vaccine hesitancy among our HHT patients may reflect the sentiments of the general South African population. Of the countries included in a 2022 global survey, South Africa was reported to have the highest rate of COVID-19 vaccine hesitancy [22].

The prevalence of COVID-19 in our HHT cohort was similar to that of the general population in the Free State Province, South Africa, over the same period (8.3% versus 7.5%) [23]. However, conclusions about disease severity and the effect of COVID-19 on epistaxis and anaemia in HHT could not be drawn from only two positive cases in this cohort. Suppressa et al. [9] found a similar prevalence, disease severity, and outcome of COVID-19 among patients with HHT compared to the general population.

Some authors hypothesise that HHT may be protective against SARS-CoV-2. Riera-Mestre et al. [7] suggested that defective angiogenesis in HHT may protect against COVID-endotheliopathy. Alternatively, stricter self-isolation among HHT patients may reduce their risk of COVID-19 infection.

The presence of HHT or AVMs, per se, should not limit access to medical treatment for COVID-19 [24]. However, certain HHT patients, like those who frequently access the healthcare system for iron infusion or blood transfusion, may be at higher risk of contracting COVID-19 [24, 25]. HHT-related complications may increase the risk for severe COVID-19 disease. These include pulmonary AVMs (PAVMs) complicated by chronic hypoxia, pulmonary arterial hypertension, or cardiac failure [25]. Available data suggest that iron status and anaemia may influence COVID-19 severity in the general population [26]. Further studies are needed to determine the influence of iron deficiency and anaemia on COVID-19 in patients with HHT.

Some of our HHT patients missed clinic appointments during the pandemic for fear of COVID-19 exposure, or because of lockdown restrictions that limited access to public transport. The European Reference Network on Rare Multisystemic Vascular Diseases (VASCERN) group found a similar preference among HHT patients for deferring elective appointments during the pandemic [24].

Telemedicine provides an attractive solution to these challenges. Gaetani et al. [27] demonstrated successful outcomes in Italian HHT patients who were followed up with virtual consultations during the pandemic. Marano et al. [28] suggested that web-mediated counselling could support patients and their families during the pandemic. Virtual consultations were not feasible in our setting as most of our patients need help to afford smartphones or access to the internet. Furthermore, telemedicine consultations are limited to patients with mild disease who do not require on-site therapies like IV iron.

The COVID-19 pandemic had a global but heterogeneous impact on mental health, with vulnerable groups (including people with pre-existing mental disorders or comorbidities and low socioeconomic status) being affected more than others [29].

Patients with HHT are at risk of mental disorders and impaired quality of life [30–34]. Epistaxis severity plays an important role [35]. Furthermore, iron deficiency anaemia independently increases the risk of psychiatric disorders [36]. Koekemoer et al. [37] recently reported reduced health-related quality of life in adults with HHT attending the UHBDC, and diagnosed depressive, anxiety, and alcohol use disorders in 4/9 (44.4%) patients.

This study provides anecdotal evidence that stress may precipitate epistaxis in some patients. Continued emotional support is important during the pandemic.

### Limitations

Because HHT is a rare disease that is probably underdiagnosed in South Africa [38], our sample size was small, which may limit the external validity of the study. The observational study design further limited the sample sizes for subset analyses. ESS and Hb data were lacking for some patients. Two deceased patients were excluded because their files could not be located. It may indicate poor record-keeping at the hospital, which should be addressed. Unfortunately, one of the deceased patients was one of the only two cases with PCR-confirmed COVID-19 disease in this study. We relied on anecdotal information about his hospitalisation for COVID-19, which may be inaccurate.

Interpretation of ESSs and Hb levels was confounded by variables unrelated to the COVID-19 pandemic. The effect of pandemic-related factors might have been demonstrated more accurately if treatment was controlled for. Implementation of the 2020 international HHT treatment guidelines [1] in the UHBDC coincided with the start of the study period. While this ensured that patients received the same first- and second-line therapies, namely nasal moisturisation and prophylactic antifibrinolytics, variation was not eliminated. Although tranexamic acid prophylaxis was prescribed in all patients, doses varied between patients. Most patients preferred petroleum jelly to moisturise their nostrils, but some used normal saline. Oral ferrous sulphate 200 mg on alternate days was prescribed for all patients, but the frequency, formulation, and IV iron dosing were inconsistent. Poor adherence to these treatments by some patients was another confounding factor.

The ESS has limitations. It does not account for IV iron infusion or other causes of anaemia or blood loss, including bleeding GI telangiectasias.

Serum ferritin would have been a more specific marker to assess for changes in iron status than Hb. However, ferritin levels were often not requested in anaemic patients, while the Hb was measured in all patients at every visit. Like Hb, other variables may also have confounded ferritin levels due to the effects of inflammation on this acute phase protein. Our single-centre study design did not allow us to demonstrate the true prevalence of COVID-19 among patients with HHT in South Africa. The prevalence of COVID-19 in the general population varied markedly between different regions of the country [23]. We could only compare the prevalence of COVID-19 in HHT patients known to the UHBDC with that of the general population in the Free State Province, which could affect the generalisability of our results.

More data is needed to guide the management of epistaxis and anaemia in HHT patients with COVID-19. This study did not address airway management, humidified oxygen, anticoagulation, thrombotic risk, possible discontinuation of antifibrinolytics and IV iron, and avoiding infectious and embolic complications of PAVMs. These aspects deserve further consideration and research.

## Conclusion

The COVID-19 pandemic had a clinically significant impact on ESSs and Hb levels at the individual level in patients with HHT in central South Africa. We identified several clinical and social variables that may influence epistaxis severity and anaemia. Nasal, rather than oral swab testing, heightened stress, missed appointments, and lockdown restrictions were noted to exacerbate epistaxis in some patients, whereas COVID-19 vaccines did not. Although patients reported worsened epistaxis with facemasks, these may be subjective perceptions, and in the absence of more extensive studies, the protective effect of masks should be weighed against their potential to worsen epistaxis. Specific strategies focusing on epistaxis and anaemia are needed to optimise the management of HHT during COVID-19 and future respiratory pandemics.

## Data Availability

Data available from the corresponding author upon reasonable request.

## Abbreviations

*ACVRL1*: Activin A receptor like type 1
AVM: Arteriovenous malformation
BMP: Bone morphogenetic protein
CAVM: Cerebral arteriovenous malformation
*ENG*: Endoglin
ESS: Epistaxis severity score
GI: Gastrointestinal
HAVM: Hepatic arteriovenous malformation
Hb: Haemoglobin
HHT: Hereditary haemorrhagic telangiectasia
NHLS LIS: National Health Laboratory Service laboratory information system
PAVM: Pulmonary arteriovenous malformation
RT-PCR: Reverse transcriptase polymerase chain reaction
TGF- beta: Transforming growth factor beta
TTCE: Transthoracic contrast echocardiography
UHBDC: Universitas Academic Hospital Bleeding Disorders Clinic
WHO: World Health Organization

## Declarations

**Ethics approval and consent to participate**

The research for this study was done in partial fulfilment of the requirements for SJK’s MMed degree (Haematology) at the University of Free State. Ethics approval was obtained from the University of the Free State Health Sciences Research Ethics Committee (UFS-HSD2021/1385/2903). The Free State Department of Health granted permission to perform the study. Written informed consent was obtained from all adult participants. Written assent was obtained for all participants under 18 years, while written consent was obtained from their parents.
